# Plant and Fungal Hepatotoxicities of Cattle in Australia, with a Focus on Minimally Understood Toxins

**DOI:** 10.3390/toxins12110707

**Published:** 2020-11-08

**Authors:** Eve M. Manthorpe, Ian V. Jerrett, Grant T. Rawlin, Lucy Woolford

**Affiliations:** 1 School of Animal and Veterinary Sciences, The University of Adelaide, Roseworthy, South Australia 5371, Australia; lucy.woolford@adelaide.edu.au; 2 Department of Jobs, Precincts and Regions, Agribio, the Centre for AgriBioscience, Melbourne, Victoria 3083, Australia; ian.jerrett@agriculture.vic.gov.au (I.V.J.); grant.rawlin@agriculture.vic.gov.au (G.T.R.)

**Keywords:** cattle, hepatotoxicity, liver, mycotoxin, pathology, plant toxin

## Abstract

Plant- and fungus-derived hepatotoxins are a major cause of disease and production losses in ruminants in Australia and around the world. Many are well studied and described in the literature; however, this is not the case for a number of hepatotoxicities with economic and animal welfare impacts, such as acute bovine liver disease (ABLD), brassica-associated liver disease (BALD) and *Trema tomentosa*, *Argentipallium blandowskianum* and *Lythrum hyssopifolia* toxicity. Additionally, significant overlap in the clinical presentation and pathology of these conditions can present a diagnostic challenge for veterinarians. This review summarizes the current and most recently published knowledge of common plant- and fungus-associated hepatotoxins affecting cattle in Australia, with a focus on the mechanisms of toxicity and distinguishing diagnostic features. Consolidation of the current understanding of hepatotoxic mechanisms in cattle provides insight into the potential mechanisms of lesser-known toxins, including cellular and subcellular targets and potential metabolic pathways. In the absence of specific etiological investigations, the study of epidemiological, clinical and pathological features of hepatotoxicity provides valuable insights into potential toxic mechanisms and is integral for the successful diagnosis and management of these conditions.

## 1. Introduction

Plant- and fungus-derived hepatotoxins are a major cause of disease and production losses in ruminants in Australia and around the world. Hepatotoxic diseases are variably described in the literature and mechanisms of toxicity, particularly in relation to domestic species, are often poorly understood. Understanding of hepatotoxins in cattle is often limited by the geographical confinement and sporadic nature of outbreaks, and in some cases, the relatively recent discovery of the disease. Hepatotoxic plants may be native or introduced as ornamental or food-producing species. Some introduced species in Australia, such as *Cestrum parqui* and *Lantana camara,* have become widespread weed species, capable of displacing native flora through allelopathic mechanisms, or through human degradation of native ecosystems [1,2,3,4]. In contrast, native species tend to be confined to certain climatic regions, allowing veterinarians to develop distinct differential diagnoses for plant-associated disease occurring in that area [4,5,6,7]. The hepatotoxic agent may be a component of the plant or be produced by fungal organisms that colonize feed. Fungal growth or contamination of feed is highly dependent on environmental factors [7,8,9,10]. As such, disease caused by mycotoxins can be seen in many areas of Australia provided that environmental conditions support growth. A common feature of these diseases is the unpalatability of the causative feed. This has clear impacts on the epidemiology of toxicity, affecting animals at times of fodder scarcity or under circumstances that reduce grazing discrimination, such as increased energy demand in gestation and lactation. The notable exception to this is toxicity caused by Cycad spp., which are selectively grazed by cattle [6]. This review will focus on the common plant and fungal hepatotoxins affecting cattle in Australia, including the epidemiology, mechanisms of toxicity, clinical presentation and clinical and gross pathology. Here, we examine features of well-understood hepatotoxins, before shifting focus to incompletely understood or unknown hepatotoxins affecting cattle. Examination of the best-characterized hepatotoxins may further elucidate mechanisms of those that are poorly understood and identify targets for future research efforts.

## 2. General Mechanisms of Toxicity

Understanding of the clinical and pathological features of hepatotoxins is facilitated by an understanding of the general mechanisms of hepatotoxicity. Hepatic uptake, biotransformation and excretion in bile are important processes to clear waste from the body; however, this function predisposes the liver to damage [11]. Hepatocytes are frequently exposed to ingested toxins, as they travel directly from the gastrointestinal tract via portal blood to the liver [12]. Many hepatotoxins exert their toxic effects after biotransformation to form reactive metabolites and free radicals [11]. In some cases, biotransformation of these molecules begins in the gastrointestinal tract, but in many cases, the liver is the first line for metabolism and formation of injurious molecules. Cytochrome P450 (CYP450) monooxygenases, a group of enzymes that are frequently implicated in toxic metabolite formation, are found in high concentrations within the endoplasmic reticulum of hepatocytes [13]. Toxic metabolites, which have increased intrinsic chemical reactivity compared to their parent compound, may be present for a short time before detoxification via binding to reduced glutathione (GSH) or cause protein haptenization or adduct formation. Therefore, cell injury by toxic metabolites is typically induced via a combination of macromolecule binding, depletion of reduced GSH, oxidative damage and lipid peroxidation [11].

Hepatotoxic agents are divided into two categories based on the predictability of toxicity. Intrinsic hepatotoxins cause predictable, usually dose-related effects, while idiosyncratic hepatotoxins produce disease rarely and unpredictably due to metabolic aberrations in certain individuals or through immunoallergic or autoimmune responses [14]. Toxins or metabolites may act directly by targeting organelles, resulting in an overwhelming direct insult and cell death [11]. Toxins that act in this manner tend to be intrinsic hepatotoxins [13]. Other toxins may cause haptenization and sensitization of the adaptive immune system and a directed response toward the target cell, resulting in non-immune-mediated, autoimmune or immunoallergic responses, as seen with certain idiosyncratic toxins [11]. The effects of a toxin also depend on the target organelle: toxins that target the nucleus are mutagenic and potentially carcinogenic, while those that target mitochondria induce cell swelling and mitochondrial-mediated cell death through translocation of cytochrome C into the cytosol [13,15]. Injury to the endoplasmic reticulum also results in the release of cytochrome C, which may induce apoptosis [13]. If the action of the toxin is more subtle, organelle damage may not be sufficient to cause cell death; however, it will induce altered signal transduction and gene expression, causing increased susceptibility to inflammatory mediators and oxidative injury [11]. This damage is likely to also cause cellular dysfunction without cell death, as seen in toxins that cause cholestasis without evidence of necrosis [14].

**Figure 1:**
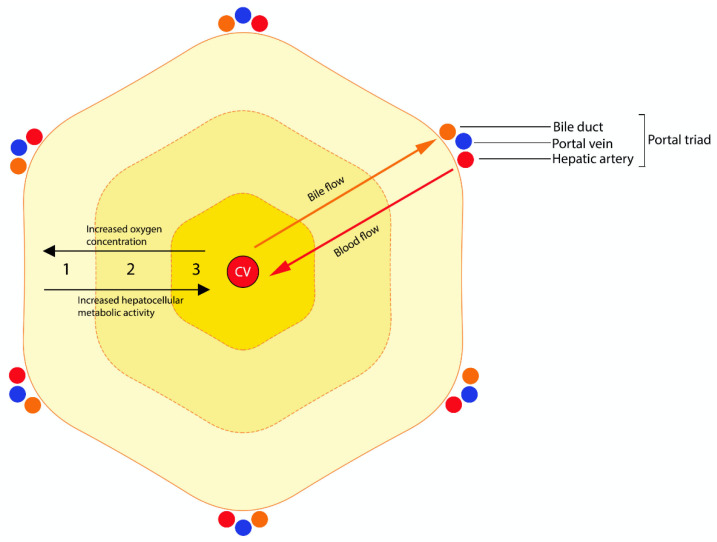
Schematic diagram of the hepatic lobule, microscopic zones and metabolic functions that relate to toxin-induced histopathological changes. The histologic zones of the hepatic lobule comprise the periportal zone (zone 1), the midzone (zone 2) and the centrilobular zone (zone 3). The centrilobular zone is furthest from the arterial blood supply, has relatively lower levels of reduced glutathione (GSH) and is rich in cytochrome p450 monooxygenases, playing a central role in the metabolism of endogenous and exogenous substances. These features confer greater susceptibility to hypoxia and oxidative injury and an increased likelihood of injury secondary to bioactivation, making the centrilobular zone the most common site of toxin-induced hepatocellular injury in the liver. The periportal zone is rich in oxygen and GSH and is the primary site of gluconeogenesis. These features confer increased regenerative capacity, resistance to oxidative injury, reduced susceptibility to hypoxia and a reduced likelihood of injury secondary to bioactivation. Toxin-induced damage to periportal hepatocytes may result from: (1) toxins that do not require bioactivation to exert toxicity, (2) toxins that require bioactivation by periportal-specific enzymes, (3) toxins that require oxygen-dependent bioactivation. The midzone has intermediate features and is most commonly injured as an extension of centrilobular injury. Abbreviations: CV, central vein. Figure modified from Brown et al. [28] and Jung and Lee [15].

The vast majority of plant and fungal hepatotoxins affecting cattle are intrinsic toxins, producing various combinations of zonal hepatocellular necrosis and biliary pathology depending on the action of the toxin. The histologic zones of the hepatic lobule (Figure 1) comprise the periportal zone (zone 1), the midzone (zone 2) and the centrilobular zone (zone 3) [15]. The centrilobular zone is particularly susceptible to injury: it is furthest from the arterial blood supply and is first affected by hypoxia [15]. It has less GSH and is rich in microsomal enzymes, such as CYP450 enzymes, and is therefore commonly affected by the toxic metabolites of this enzyme system [15]. In contrast, the periportal zone is rich in periportal-specific enzymes, GSH and oxygen and is the primary site of gluconeogenesis [15]. These features confer an increased regenerative capacity and reduced susceptibility to hypoxic injury but an optimal environment for oxygen-dependent bioactivation [15]. It has been suggested that periportal necrosis may result in more severe disease due to reactive oxygen species formation and reduced hepatic regenerative capacity [16].

Periportal hepatocellular damage is an uncommon lesion and causative toxins must have one of the following characteristics: (1) they do not require CYP450 bioactivation to exert toxicity, (2) they require bioactivation by periportal-specific enzymes or (3) they require oxygen-dependent bioactivation. Ferrous sulfate [16], yellow phosphorus [17] and allyl formate [18,19] are known to cause periportal hepatocellular injury in humans. Ferrous sulfate and yellow phosphorus toxins do not require bioactivation to exert toxicity, causing periportal damage due to the direct cytopathic effect of the highest concentration of toxin carried by portal blood flow [17,20,21,22]. In contrast, allyl formate requires bioactivation to acrolein by alcohol dehydrogenase, an enzyme localized within periportal hepatocytes [18,19]. Other drugs and xenobiotics, such as acetic acid, albitocin, synthalin, alloxan, pemphigus vulgaris endotoxin and mesalazine are known to cause periportal necrosis in humans, but the mechanisms are not well understood [14,23,24,25]. Examples in ruminants are limited to *Myoporum* spp. poisoning and acute bovine liver disease (ABLD), which are minimally understood and discussed later [26,27].

## 3. Clinical Findings

Although variation exists among plant- and fungal-associated hepatotoxicities, there is a significant overlap in the clinical presentations of these diseases in cattle. Familiarity with these general characteristics is important in order to highlight the unique features of each toxin. Acute hepatotoxicity is a common presentation, resulting in a combination of inappetence, lethargy, reduced milk production, jaundice, secondary (hepatogenous) photosensitization and hepatic encephalopathy. If there is minimal access to shade and high ultraviolet radiation exposure, photosensitization may develop within hours of toxin ingestion, characterized by erythema and exudative skin lesions progressing to ulceration and necrosis of unpigmented regions [28,29,30]. Hepatic encephalopathy typically occurs in the terminal stages of disease and is characterized by behavioral derangements ranging from depression, ataxia and recumbency to aggression, mania, muscle tremors and terminal convulsions [28,30,31].

Chronic hepatotoxicity is the primary presentation for a number of hepatotoxins including pyrrolizidine alkaloids [31], phomopsins [32], sporidesmin [33] and aflatoxin B1 [8,34]. Chronic hepatic or cholestatic injury frequently results in ill-thrift, reduced feed intake and reduced weight gain and thus should be considered a differential diagnosis in cases of unexplained poor production. The development of jaundice, hepatic encephalopathy and/or photosensitization may occur in some cases, allowing clinical localization to the hepatobiliary system. Death without prior clinical signs (sudden death), may result from the ingestion of many hepatotoxins if large enough quantities are consumed. Sudden death in a large proportion of affected animals may suggest the involvement of a particularly potent toxin such as microcystin produced by cyanobacteria [35], carboxyparquin produced by *Cestrum parqui* (green cestrum) [1] or ABLD toxin [36].

## 4. Clinical Pathology

Biochemical changes are variably described for hepatotoxic diseases in cattle, likely due to the nonspecific nature of these findings. Where available, biochemical changes follow expected patterns of derangements for the action of the toxin. The majority of hepatotoxins discussed here cause damage to both hepatocytes and cholangiocytes, causing elevations in both hepatocellular and cholestatic enzymes. As a marker of functionality, hyperbilirubinemia is seen in any case where hepatic function is sufficiently compromised. Toxins that target hepatocytes, such as cycasin from Cycad spp. [37], carboxyparquin from green cestrum [1] and furanosesquiterpenoid essential oils from *Myoporum* spp. [26], cause marked elevations in hepatocellular enzyme activities such as aspartate transaminase (AST) and glutamate dehydrogenase (GLDH), while elevations of cholestatic enzyme activities, although frequently present, are typically at lower amplitudes than hepatocellular enzymes [1,26,37]. Aspartate transaminase is not a specific marker of hepatocellular damage and should be interpreted in light of other analytes, such as creatinine kinase (CK), in cases of prolonged recumbency. In contrast to dogs and cats, hepatocyte alanine aminotransferase (ALT) concentrations are low in ruminants, offering no diagnostic specificity for liver disease in these species [38]. Toxins that target cholangiocytes, namely sporidesmin [39] and aflatoxin B1 [8,34], cause marked elevations in cholestatic enzyme activities such as gamma-glutamyl transferase (GGT) and alkaline phosphatase (ALP) [34,40]. Acute cases have the potential to cause elevations in hepatocellular enzyme activities; however, the elevation is commonly less marked than that of the cholestatic enzymes [40]. For example, acute cases of pithomycotoxicosis in New Zealand have been shown to cause elevations of GGT activity, ranging from 2000 to 4000 IU/L (expected range 0–36 IU/L) and elevations of GLDH activity, ranging from within reference to 2000 IU/L and rarely more (expected range 8–41 U/L) [40].

Chronic biochemical changes reflect reduced functional liver capacity. Reduced carbohydrate and nitrogen metabolism may cause reduced blood urea nitrogen (BUN) and ultimately ammonia intoxication, the primary factor contributing to hepatic encephalopathy [4,28,41]. There may also be reduced production of prothrombin and albumin, resulting in hypoproteinemia and an antithrombotic state [4]. Normocytic, normochromic anemia (anemia of chronic disease) and hyperbilirubinemia may also be observed [31]. Gamma-glutamyl transferase serum levels are slow to increase and may remain high for several months after the initial insult; however, diseases that have a prolonged clinical time course, such as pithomycotoxicosis and pyrrolizidine alkaloidosis, may not demonstrate elevated enzyme activities at the time of clinical presentation [31,33]. Increased serum bile acids are highly sensitive for hepatobiliary dysfunction and may be clinically useful when clinical signs or enzyme changes are equivocal [38,42].

## 5. Pathological Features

Toxin-induced injury can be divided into the following broad categories: cholestatic injury, necroinflammatory injury, steatosis, fibrosis and cirrhosis, and vascular lesions [14].

**Figure 2:**
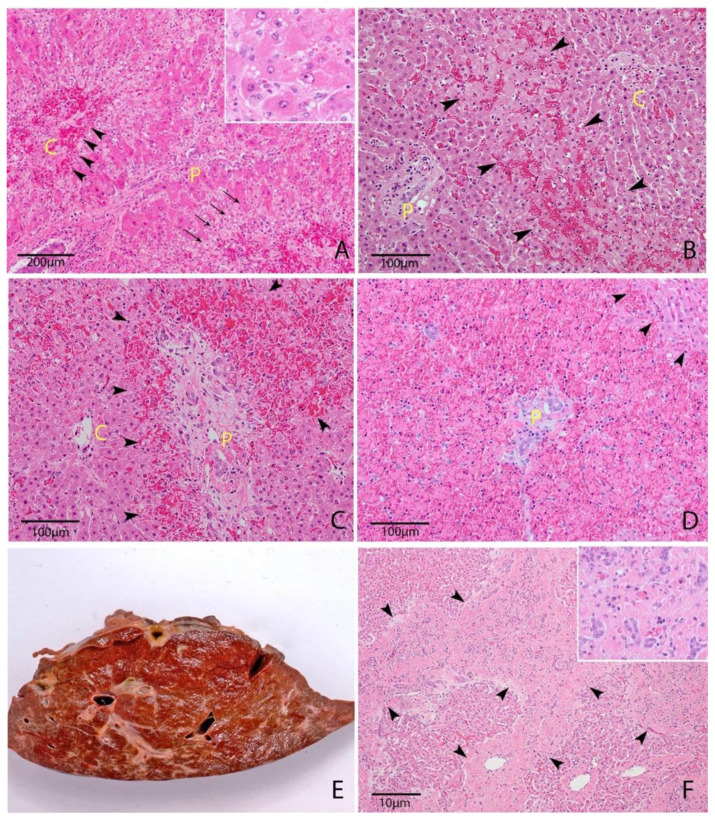
(**A**–**D**) Hematoxylin and eosin-stained sections of bovine livers affected by acute toxicity, demonstrating zonal hepatocellular necrosis. (**A**) Centrilobular hepatocellular necrosis, 5-month-old Friesian calf, sudden death due to concurrent pyrrolizidine alkaloid and copper toxicity. There is acute coagulative necrosis and loss of centrilobular hepatocytes with hemorrhage (arrowheads) and vacuolar change to midzonal hepatocytes (arrows) (10×). Inset: midzonal to periportal hepatocytes show microvesicular vacuolation consistent with hydropic degeneration and anisocytosis and anisokaryosis consistent with pyrrolizidine alkaloid toxicity (40×). (**B**) Midzonal hepatocellular necrosis, adult female, unknown breed, acute *Lythrum hyssopifolia* (lesser loosestrife) poisoning. There is acute coagulative necrosis and loss of hepatocytes with hemorrhage confined to the midzone (arrowheads) (20×). (**C**) Periportal hepatocellular necrosis, adult female Angus, acute bovine liver disease. There is acute coagulative necrosis and loss of hepatocytes with hemorrhage confined to the periportal zone (arrowheads) (20×). (**D**) Massive hepatocellular necrosis, adult female Friesian, acute bovine liver disease. There is acute coagulative necrosis and loss of hepatocytes with hemorrhage spanning all three zones of the lobule, a small number of centrilobular hepatocytes remain (arrowheads) (20×). (**E**,**F**) Bovine livers affected by chronic pithomycotoxicosis, demonstrating chronic hepatotoxic injury. (**E**) Liver, left lobe. There is a marked portal and bridging fibrosis and brown-green discoloration of the liver due to cholestasis. (**F**) Liver, left lobe. There is a marked portal and bridging fibrosis (arrowheads) and marked biliary hyperplasia (4×). Inset: hyperplasia of biliary profiles surrounded by mature collagen (40×). Abbreviations: P, portal region; C, central vein.

Cholestatic injury occurs when the toxic agent targets cholangiocytes, resulting in reduced bile flow [14]. In ruminants, this is typified by facial eczema (pithomycotoxicosis). Macroscopically, there is the prominence of superficial bile ducts, obstruction with bile plugs or inspissated debris and distension of the gallbladder (Figure 2) [41,43]. Acute or subacute cases typically demonstrate a swollen, friable, pale and yellow-green discolored liver reflecting bile accumulation [3,34,41,43,44,45]. These lesions are reflected microscopically by canalicular distension with bile, bile pigment within hepatocytes and Kupffer cells and marked ductular reaction (biliary hyperplasia (Figure 2)) [43]. There may also be portal infiltration with inflammatory cells, typically mononuclear cells, variable cholecystitis and gallbladder wall edema [43].

Necroinflammatory injury is characterized by hepatocellular degeneration and necrosis, commonly zonal, with varying degrees and classes of inflammation (Figure 2) [14]. In these cases, macroscopic findings may include mild hepatomegaly, pale-brown discoloration of the liver and an enhanced reticular pattern caused by zonal hepatocellular necrosis, loss and hemorrhage [1,26,46,47,48]. Panlobular (massive) necrosis may occur in particularly severe cases (Figure 2). This type of injury is seen in carboxyatractyloside [49], cycasin [37,50], carboxyparquin [1], punicalagin [47,51,52], *Argentipallium blandowskianum* [53], *Trema tomentosa* [54] and *Myoporum* spp. toxicity [26]. Attempted regeneration, in the form of mild to moderate ductular reaction, is a common feature [47].

When acute cholestasis and necroinflammatory lesions occur together, it is termed cholestatic hepatitis and represents a mixed type of hepatic injury [14]. This is commonly seen in pyrrolizidine alkaloid [4,30], aflatoxin B1 [8,34], phomopsin [32,55] and *L. camara* toxicity [45]. Chronic injury to any region of the liver will eventually result in fibrosis (Figure 2) and possibly nodular regeneration (cirrhosis) [14]. The location of fibrosis (centrilobular or portal) is dependent on the initial target of the toxin [14]. Vascular lesions are characteristic of a handful of toxins in ruminants, including pyrrolizidine alkaloids [4], sporidesmin [40,43] and *A. blandowskianum* [53], and are discussed further individually. Another common feature of hepatotoxicity is the presence of widespread serosal or subcutaneous petechial hemorrhages [43,47,56]. The exact mechanism for this is unclear but likely involves uncontrolled consumption of clotting factors in the injured liver [28]. This may be exacerbated by the diminished synthesis of clotting factors with short half-lives (factors V, VII, IX and X), diminished clearance of coagulation byproducts, abnormal fibrinogen synthesis and abnormal platelet function due to metabolic disturbances and accumulation of fibrin degradation products [28].

## 6. Toxins with Known Mechanisms of Toxicity

### 6.1. Direct-Acting Plant Toxins

#### Carboxyatractyloside

*Xanthium strumarium* (Noogoora burr) is an annual herb of the Asteraceae family, possibly introduced to Australia with cotton seeds from southern USA before 1880 [57]. It has now become a weed throughout large parts of Australia, preferring areas that experience occasional flooding [57]. The toxic component, carboxyatractyloside, is found only in cotyledons (seed leaves) and burrs; therefore, toxicity commonly occurs when spring or summer rainfall allows widespread germination and cotyledons become prevalent in the infested area (Table 1) [57]. Once the first true leaves have grown, the plant is no longer hazardous [57]. Carboxyatractyloside, a diterpenoid glycoside, is water-soluble and readily absorbed from the gastrointestinal tract [49]. It requires no biotransformation to exert toxicity and can cause clinical disease anywhere from a few hours to three days after ingestion (Table 2) [49].

Hepatotoxicity is due to competitive inhibition of the adenine nucleoside carrier in oxidative phosphorylation, which blocks adenosine triphosphate (ATP) transport from mitochondria into cytosol, depleting the cellular energy source (Table 2) [49]. The lack of ATP and mitochondrial pore leakage ultimately lead to cell death and necroinflammatory injury targeting centrilobular hepatocytes (Table 3) [54]. The extent of necrosis depends on dosage, and in lethal toxicity, there is typically midzonal vacuolation and centrilobular necrosis, but hepatocellular injury can be panlobular [54]. The toxin does not have cumulative effects, suggesting that the metabolism and detoxification of carboxyatractyloside are rapid [58]. In addition to clinical signs of acute hepatotoxicity, gastrointestinal irritation is a notable feature characterized by abdominal pain, anorexia and hypersalivation [54,58]. Evidence of gastrointestinal irritation is seen at postmortem [49]. Histologically there may also be evidence of acute nephrosis with mild to moderate tubular degeneration [49]. The diagnostic features of carboxyatractyloside toxicity are summarized in Table 1.

### 6.2. Plant Toxins Requiring Bioactivation

#### 6.2.1. Pyrrolizidine Alkaloids

Pyrrolizidine alkaloids (PA) are a group of plant metabolites composed of a necine base, one or more necic acids and two fused rings joined by a single nitrogen atom (the pyrrolizidine core) [59]. There are several necine bases including platynecine, retronecine, heliotrodine and otonecine [60]. The most abundant and toxic of these are the retronecine-, heliotridine- and otonecine-type PA [60]. Pyrrolizidine alkaloids are found in over 6000 plant species, primarily in the families Boraginaceae, which includes the genus *Heliotropium;* Compositae, which includes the genera *Senecionae* and *Eupatoriae*; and Leguminosae, which includes the genus *Crotalaria* [57,61,62].

There is a significant variation in species susceptibility to pyrrolizidine alkaloids. Pigs are the most susceptible, followed by poultry, then horses and cattle and finally sheep and goats [57]. Being grazers, cattle, horses and sheep are most likely to be affected, despite their relative resistance [57]. It is reported that young animals are more susceptible to pyrrolizidine alkaloid toxicity than adults; however, this is extrapolated from field reports and the mechanism is not well understood [59,63]. The toxicokinetics and toxicodynamics of PA are briefly summarized in Table 2. After ingestion, PA are absorbed from the gastrointestinal tract into portal circulation [61]. Approximately 80% are excreted unchanged into urine and a small amount is excreted in feces and milk [61]. To exert toxicity, bioactivation must occur. The primary site of metabolism is the liver; however, the lungs (club, formerly Clara, cells) and to a lesser extent, the kidneys (proximal convoluted tubular epithelium), play a role in the metabolism of these compounds [59,64].

Metabolism occurs via three routes: hydrolysis by esterases to produce necines and necic acids; N-oxidation to produce pyrrolizidine alkaloid N-oxides (PANO); and oxidation to produce dehydropyrrolizidine alkaloids (DHPA, ester pyrroles), the primary toxic components [59]. Oxidation is primarily performed by CYP450 monooxygenases; therefore, lesions are most pronounced in the centrilobular regions of hepatic lobules [59]. Necines and necic acids are nontoxic and can be excreted. Pyrrolizidine alkaloid N-oxides can also be conjugated and excreted; however, if levels of PANO overwhelm conjugation pathways, they can revert back to PA and thus undergo oxidation to form DHPA [59]. Dehydropyrrolizidine alkaloids are highly reactive and hepatotoxic, binding with proteins containing sulfur, nitrogen and oxygen and forming adducts at the site of formation [59]. Additionally, DHPA may undergo hydrolysis to form secondary toxic metabolites, pyrrolic alcohols [59]. These are less reactive, more persistent and able to penetrate the nucleus and form DNA-crosslinks and are therefore responsible for mutagenic and carcinogenic effects of PA [59,65,66]. Long-living pyrroles may also travel to the lungs and heart where they can cause damage to macromolecules [59,67]. Pulmonary lesions are well described in humans and rats but are not commonly noted in ruminants [59,67]. Lipophilic metabolites also have the potential to cross the placenta and have been reported to cause hepatotoxicity in fetuses [61,68]. After production of the pyrroles, the next detoxifying mechanism relies on conjugation with soluble cytoplasmic molecules, such as GSH [59]. Pyrrole-GSH adducts can then be excreted into bile or sinusoidal blood for clearance from the liver [59]. Differences in species susceptibility can therefore be explained, at least in part, by the action of the CYP450 monooxygenases and the availability of GSH.

Pyrrolizidine alkaloid toxicity commonly causes signs consistent with chronic hepatic and cholestatic disease, with emaciation occurring over several weeks [31]. Diarrhea and tenesmus leading to rectal prolapse are also commonly seen [30,31,34]. Pathologic changes have been well described and are consistent with chronic cholangiotoxic disease with the addition of periportal to midzonal megalocytosis and, less commonly, renal epithelial megalocytosis [4,30]. This finding results from pyrroles, which cause inhibition of mitosis without inhibition of DNA production, and parenchymal injury, which provides a stimulus for regeneration [59]. Vascular lesions are a notable feature in cattle, characterized by fibrosis around hepatic veins, compressing and sometimes obliterating the affected vessel [4]. In humans, this is known as veno-occlusive disease and occurs due to the toxic effects of metabolites on endothelial cells of hepatic venules [59,69]. Acute toxicity is uncommon but has been reported experimentally, resulting in centrilobular hepatocellular necrosis [4]. Megalocytosis is less common because hepatocytes have not had time to increase in size prior to the death of the animal [4]. The diagnostic features of PA toxicity are summarized in Table 1.

#### 6.2.2. Cycasin

Cycads are a group of primitive, palm-like plants belonging to the order Cycadales, composed of three families: Cycadaceae, Strangeriaceae and Zamiaceae [70]. In Australia, the genera *Bowenia, Cycas, Lepidozamia* and *Macrozamia* are responsible for livestock losses and are restricted to tropical and subtropical regions [5,6,57]. Cycads are often selectively grazed by livestock, with certain individuals becoming “addicted” to the plants and passing the habit to others in the group (Table 1) [6]. Cycad ingestion results in two clinical syndromes: acute gastrointestinal and hepatic disease (Table 1), and a neurological syndrome [5,6]. The gastrointestinal condition is characterized by dysentery, anorexia and liver injury and occurs shortly after the ingestion of plant material (Table 1) [50]. If access to the plant continues, animals that survive the acute presentation are likely to progress to the neurologic syndrome within a few weeks to a month [37,71]. This is characterized by ataxia and proprioceptive hindlimb deficits, progressing to recumbency and death [37,71,72].

Cycasin and macrozamin, the major glycosides found in cycads, are converted to the aglycone methylazoxymethanol (MAM) by β-glucosidases in the gastrointestinal tract (Table 2) [54,70]. Cycasin itself is a potent mucosal irritant and contributes to the gastrointestinal pathology observed in acute cases [70]. After absorption, MAM is further metabolized by hepatic microsomal enzymes to form methanol and formic acid [54,73,74]. These DNA-methylating species are responsible for hepatotoxicity (Table 2) [54,73,74]. Another two toxins have been isolated from cycads: β-methylamino-l-alanine and an unidentified high-molecular-weight compound, both of which are neurotoxins [75]. These toxins are in all parts of the plant but are in the highest concentrations in the seeds and roots [70,75]. It is by these mechanisms that cycads are neurotoxic, carcinogenic, mutagenic, teratogenic and hepatotoxic [70,75,76,77]. Toxicity appears to affect many organ systems including, most obviously, the liver, gastrointestinal tract and central nervous system, but also the kidneys and lungs [77]. Pathological findings reflect acute necroinflammatory hepatotoxicity targeting centrilobular hepatocytes, hemorrhagic necrosis of the small intestinal and abomasal mucosa and acute renal tubular injury (Table 1) [37,50,54]. Chronically affected livers often demonstrate centrilobular to bridging fibrosis, cholestasis, fatty change and hepatocellular megalocytosis, caused by the mitoinhibitory effects of the metabolites [28,50].

### 6.3. Direct-Acting Mycotoxins

#### 6.3.1. Phomopsins

Ingestion of lupin stubble contaminated with the saprophytic fungus *Diaporthe toxica* (formerly *Phomopsis leptostromiformis*) causes a condition known as lupinosis in ruminants [10]. It should not be confused with lupin toxicity caused by the consumption of quinolizidine alkaloids from the plant [78]. Lupins are well adapted to sandy soils and are used extensively as a food source for cattle and sheep in Australia [79]. *Diaporthe toxica* first acts as a parasitic fungus on the green plant, with spores landing on the surface, germinating and then penetrating the inner stem [54]. When the plant begins to senesce, *D. toxica* becomes a saprophytic fungus presenting as dark lesions on the stem [7,80]. Two spore types are responsible for the transmission of infection: pycnidiospores, which are transported by water and disperse in a small radius, and ascospores, which are transported by wind and can travel up to 2 km [7]. In general, stubble tends to become unsafe after a period of rainfall, particularly post-summer rainfall and toxin will continue to accumulate until the end of autumn, resulting in the highest toxicity in late autumn (Table 1) [7,10]. Under laboratory conditions, the ideal temperature for toxin production in liquid media is 25 °C [81]. The ideal relative humidity is not known but is likely to be high due to increased toxicity post-rainfall [81].

Toxicity is caused by the secondary metabolites of the fungus, phomopsins A through E, which are a group of hexapeptide macrocyclic mycotoxins [79,82]. Phomopsin A is the most toxic and abundant metabolite and is often accompanied by small quantities of phomopsin B, which has potential toxicity two to five times less than phomopsin A [79,82,83]. The occurrence of phomopsins C, D and E have been reported but are less common [82]. Unlike pyrrolizidine alkaloids, no bioactivation is required to exert toxicity; in fact, phomopsins are rapidly metabolized to less toxic components in the liver [81]. Toxicity is due to the ability of phomopsins to bind tubulin, preventing the formation of new microtubules and depolymerizing existing microtubules (Table 2) [78,81]. Microtubules are essential for normal cell function, intracellular transport and mitosis [81]. Phomopsins also have the potential to impair mitochondrial respiration (Table 2) [81]. Both of these toxic effects are time and concentration-dependent and culminate in direct cytotoxicity and mitosis inhibition [78]. It has been suggested that phomopsins can interact with DNA resulting in chromosomal aberrations, supported by the demonstration of carcinogenic effects in rats [81,84].

In cases of lupinosis, the liver is the main target organ due to its role in metabolism; however, other organs may demonstrate lesions, including the kidneys, adrenal glands, pancreas, rumen and reticulum (Table 2) [81]. Lupinosis in cattle results in two distinct syndromes. The “fatty-liver syndrome” is seen primarily in late-pregnant or recently calved cows and is not a result of the direct action of phomopsins but a sequela of toxin-induced inappetence and subsequent ketosis [32]. This form is relatively common and is characterized by midzonal to massive hepatocellular steatosis and fatty degeneration of renal tubular epithelium, resulting in acute clinical deterioration and death [32,55]. The “cirrhotic liver syndrome,” caused by the chronic toxic effects of phomopsins, is more common in sheep than cattle and is summarized in Table 1. It is characterized by chronic hepatocellular loss, replacement fibrosis and impaired mitotic activity resulting in progressive hepatic shrinkage [32,55]. Histologically, there is increased mitotic activity and hepatocyte death [54]. Mitoses are frequently abnormal and appear arrested in late metaphase [54]. Remaining hepatocytes may appear swollen with granular cytoplasm and occasional intranuclear pseudoinclusions [54].

#### 6.3.2. Sporidesmin

Sporidesmin is a mycotoxin produced by *Pithomyces chartarum* on infected Perennial Ryegrass pastures, resulting in facial eczema (pithomycotoxicosis) [8]. The ideal conditions for sporulation occur around late summer and early autumn, attributable to maximum temperatures between 20 and 25 °C, overnight temperatures higher than 14 °C and very high humidity (Table 1) [39]. Ingestion of sporidesmin causes chronic liver damage and severe photosensitization, which does not occur for at least a week, commonly several months after initial exposure (Table 1) [8,85]. Mortality rates can be high; however, in general, greater losses occur from increased culling due to skin lesions or carcass condemnation [33]. Cattle that apparently recover from photosensitization are at increased risk of liver insufficiency during periods of high energy demand [33]. The vast majority of sporidesmin produced by *P. chartarum* is sporidesmin A, although sporidesmins B through J are also produced in smaller quantities [39]. Sporidesmin is rapidly absorbed from the intestines and concentrated in the liver and biliary system [39]. Excretion primarily occurs through bile, with only a small amount excreted in urine [39]. Toxicity appears to be due to the cyclic reduction of sporidesmin, a reaction that is strongly catalyzed by copper and results in the formation of a superoxide radical [39]. Zinc inhibits the absorption of copper and activates superoxide dismutase and is therefore known to be partially protective [39]. The toxicokinetics and toxicodynamics of sporidesmin are summarized in Table 2.

Sporidesmin is particularly toxic to bile canalicular membranes with macroscopic and microscopic changes characterized by acute to chronic cholestatic injury [39,41,43]. Vascular occlusive lesions are a key finding in sheep and goats and are occasionally seen in cattle. This is characterized by fibroplasia in the tunica intima and media proliferating into the lumen of small vessels, or large branches of the portal vein and hepatic artery on the side that is nearest the damaged bile duct [40,43]. In chronic cases, there is often left liver lobe atrophy and fibrosis [41,43]. The exact mechanism for this is unclear but may result from the differential flow of portal blood from one area of the intestine to a certain lobe of the liver (portal streaming) [28]. The diagnostic features of facial eczema are summarized in Table 1.

#### 6.3.3. Amatoxins

Amatoxins are potent hepatotoxins found in many mushroom species of the genera *Amanita, Lepiota* and *Galerina* [86]. A notable example is *Amanita phalloides* (death cap), which commonly causes lethal toxicity in human beings [86]. Although not commonly associated with toxicity in ruminants, amatoxins are included here because they provide an example of extremely potent mycotoxins. Amatoxin-producing mushrooms demonstrate confined growth in Australia: *Amanita muscaria* is found only in small pockets of south-eastern Australia, and *Amanita phalloides* is found only in Melbourne and the northern parts of the Australian Capital Territory [57]. The amatoxins are composed of at least nine different compounds; of these, α-amanitin is the most abundant and potent, with an intraperitoneal lethal dose 50 (LD_50_) of 0.4 to 0.8 mg/kg in white mice, and an estimated oral LD_50_ of 0.1 mg/kg in humans [46,87,88]. These toxins are incredibly stable; resistant to cooking, drying and freezing as well as acid and enzyme degradation [89]. After ingestion, they are rapidly absorbed from the gastrointestinal tract and distributed to the liver and kidneys (Table 3) [90]. Amatoxins do not undergo metabolism and are excreted unchanged in the urine [86].

The mechanism of toxicity is primarily due to inhibition of RNA polymerase II activity within the nucleus of target cells [91]. Through this mechanism, amatoxins inhibit mRNA and protein synthesis, which ultimately leads to the death of the cell (Table 2) [91]. Other mechanisms such as p53- and caspase-3-induced apoptosis, TNF-α mediated damage and the formation of reactive oxygen species, are likely to be involved but are incompletely understood [86]. In humans, amatoxin-induced liver injury is characterized by centrilobular to massive hemorrhagic hepatic necrosis and fatty degeneration [86]. Renal lesions are common and include cortical hemorrhage, renal tubular necrosis and marked hyaline cast deposition in tubules [92]. Given the restricted growth of these mushrooms in Australia, toxicity in grazing animals is uncommon and there are currently no case reports of amatoxin toxicosis in Australian cattle. Sporadic case reports in the United States suggest that amatoxin-related lesions in cattle are similar to those in humans, and the toxicokinetics and toxicodynamics are likely to be similar [46]. The diagnostic features of amatoxin toxicity are summarized in Table 1.

### 6.4. Mycotoxins Requiring Bioactivation

#### Aflatoxins

Aflatoxins are a group of chemically similar compounds primarily produced by *Aspergillus flavus* and *Aspergillus parasiticus* [9,93]. Many aflatoxins have been identified; however, aflatoxins B1, B2, G1 and G2 predominate [8,9,93]. Summer cereals, particularly maize and peanuts, are susceptible to aflatoxin contamination [9]. In Australia, this is primarily a concern in non-irrigated peanut crops in south-east Queensland [8,9]. In healthy crops, natural plant defenses inhibit the growth of the fungus; however, in high temperatures and low moisture, damage to seed kernels can allow the fungus to invade (Table 1) [9]. Although significant preharvest contamination is a concern for these crops, contamination of other feedstuffs tends to occur due to high moisture content during storage (Table 1) [9]. Growth of fungus and aflatoxin production are dependent on a number of factors, but relative humidity and temperature appear to be the most important [93]. *Aspergillus flavus* can grow in water activities (w_a_) between 0.91 and 0.99 and temperatures between 15 and 37 °C. Production of aflatoxin is in a narrower range, occurring in temperatures between 25 and 37 °C, and limited by an active water level of 0.94 w_a_ or lower [93].

Aflatoxin B1 (AFB1) is the most toxic metabolite and produces dose-dependent effects [8]. It has a low molecular weight and is lipophilic, allowing rapid and passive absorption from the duodenum, a process that occurs more rapidly in young animals [94]. Aflatoxin B1 can reversibly bind albumin, resulting in an equilibrium of bound and unbound toxin in the blood, with unbound fractions able to pass freely into tissues [94]. It accumulates in many tissues: the largest amount accumulates in the liver, followed by the kidneys, bone marrow and lungs, and then the brain, muscle and adipose tissue [94]. It undergoes biotransformation in the liver, kidney and small intestine to produce a number of toxic and carcinogenic metabolites [94]. In the liver, CYP450 enzymes activate AFB1 to form epoxide, an alkylating agent capable of binding DNA, RNA and proteins [95]. This results in reduced production of these components in the affected cell, disruption of normal metabolism, cell death or mutagenesis [94]. Cytochrome P450 metabolism in the liver also produces the metabolite M1, which has equivocal toxicity to B1 and is excreted in milk, allowing detection in milk two to four days after ingestion [44,94,95]. Aflatoxin B1 toxicokinetics and toxicodynamics are summarized in Table 2.

Chronic aflatoxicosis is the primary presentation, characterized by chronic cholangiotoxicity, with the addition of hepatocellular megalocytosis and extrahepatic cholestatic changes such as loss of fecal pigmentation [34,44,96]. Acute toxicosis is uncommon but has been reported in susceptible cattle with limited access to other food sources [97]. In these cases, there is mixed cholestatic and hepatocellular injury, characterized by extrahepatic cholestasis, biliary hyperplasia, mild neutrophilic hepatitis, periportal megalocytosis and vacuolar degeneration and necrosis of centrilobular hepatocytes [97]. The diagnostic features of aflatoxicosis are summarized in Table 1.

## 7. Hepatotoxicities Caused by Toxins with Unidentified Mechanisms or Unidentified Toxins

### 7.1. Toxins with Unidentified Mechanisms

#### 7.1.1. Lantana Camara

*Lantana camara* is an ornamental shrub of the Verbenaceae family, found in many parts of the world [3,4]. It is not native to Australia and is one of five weeds covering large parts of pastures in central Queensland and the dry rainforest in the Macleay River in New South Wales [3,4]. Spread has been promoted by burning, biomass removal and soil scarification [3]. Triterpenoids are responsible for toxicity: Lantadenes A, B, C and D are the primary constituents [3,98]. Lantadene A (LA) is the most toxic and abundant component of leaves and is therefore the most clinically relevant [3]. The toxic actions of lantadenes are not well understood; however, it is suspected that metabolites may play a role. Sharma et al. [99], found that LA and LB are metabolized to reduced forms, reduced LA (RLA) and reduced LB (RLB), and two unidentified metabolites, M1 and M2 in the cecum (Table 3). Later, RLA was shown to inhibit state-3 respiration in rat livers in a concentration-dependent manner, while LA inhibited state-3 respiration only at high doses [100]. RLA also impacted the mitochondrial membrane potential and reduced ATP concentrations [100]. Both LA and RLA inhibit mitochondrial respiration through inhibition ATP synthase (Table 3) [100]. This study suggests that the toxicity of lantadenes is due, at least in part, to the inhibition of mitochondrial respiration, and that RLA may be more toxic than the parent compound [100].

Toxicity reduces neural impulses for ruminal contraction, causing inappetence, constipation and the dry fecal material seen at postmortem, as well as concentration of lantana toxins in the rumen [98]. The subsequent course of disease depends on the quantity of foliage eaten but commonly includes the development of severe photosensitization within 24 to 48 h [3,45]. Recovery from the first exposure confers some resistance to subsequent exposures [98]. Macroscopic and microscopic findings are consistent with acute cholestatic injury, with the frequent addition of hepatocellular megalocytosis [3,45]. The cause of this is unknown but is possibly due to enlargement of the rough endoplasmic reticulum [45]. Swollen and congested kidneys may also be seen if death occurs more than five days post-ingestion, characterized microscopically by degeneration and necrosis of proximal tubular epithelium and occasional cystic distension of tubular lumens [45]. The diagnostic features of *L. camara* toxicity are summarized in Table 3.

#### 7.1.2. Carboxyparquin

*Cestrum parqui* L’herit, also known as green cestrum, is a perennial shrub of the Solanaceae family, which has become a common weed in areas of south-east Queensland, eastern New South Wales, Victoria and high rainfall areas of South Australia [1,2]. It is native to South America and was introduced to Australia as a garden plant, which normally grows along watercourses and in non-crop areas [1,101]. It is known to cause hepatoxicity and sudden death in ruminants, particularly cattle, but is unpalatable and generally avoided unless there is low feed availability (Table 3) [1,101]. Spraying and cutting the plant, without removing debris, may increase the risk of stock losses as the wilted leaves are more palatable than fresh leaves [101]. Carboxyparquin, a diterpenoid glycoside and hepatotoxic agent, has been isolated from the leaves and stems of the plant but the toxicodynamics and toxicokinetics are not well understood [2]. Outbreaks are relatively uncommon, highly irregular and often restricted to only a few individuals within a herd with high mortality rates [1]. Mortalities may be acute, occurring within 24 h when large quantities have been consumed [1]. Gross and microscopic changes are characterized by acute necroinflammatory toxicity targeting centrilobular hepatocytes [1]. The diagnostic features of carboxyparquin toxicity are summarized in Table 3.

#### 7.1.3. Punicalagin

Punicalagin is a hydrolysable vegetable tannin, which is present in *Terminalia oblongata* (yellow-wood) [51] and *Punica granatum* Linne (pomegranate) [102]. Punicalagin is a high-molecular-weight, hydrophilic polyphenolic molecule [103]. It has been studied in detail for its antioxidant effects in humans and has been found to be safe for consumption at normal dietary doses [103]. The metabolism of punicalagin is not well understood: a study by Cerda et al. [104] found that metabolism appears to occur in two stages in rats. The first stage occurs during the first three weeks of ingestion and is predominated by hydrolysis, which is followed by the adaptation of intestinal microflora and production of different metabolites after three weeks of ingestion [104]. Additionally, after oral administration in rats, five metabolites have been isolated in the liver and kidneys, suggesting these organs play a role in metabolism [105]. Polyphenols are known to be cytotoxic at high concentrations, causing reduced human neoplastic cell growth by binding cell proteins, particularly cell membrane proteins [103,106]. It is therefore possible that this mechanism plays a role in the toxicity of punicalagin [103].

*T. oblongata* is a native Australian deciduous tree of the family Combretaceae, which grows over a large area of eastern Queensland [51,57]. It contains two toxic components: punicalagin, responsible for hepatotoxicity, and terminalin, a nephrotoxic tannin [51,52]. Pathologic lesions have been well described in mice and are characterized by dose-dependent renal tubular necrosis and centrilobular necroinflammatory injury [51,52]. The two toxins contribute to different disease courses related to duration and severity of exposure: acute intoxication is likely to result in liver failure, while chronic cases may terminate in renal failure [51]. *Punica granatum* L. is a fruit-bearing tree that was introduced to Australia for the production of human food and drink [102]. Punicalagin is particularly concentrated in pomegranate rind [102] and along with other reactive phenols, is responsible for the astringent flavor of the skin and white pulp [47]. The use of pomegranate primarily in human food production means that it is not commonly implicated in the toxicity of ruminants. Additionally, rodent studies have shown the toxic dose of pomegranate-derived punicalagin to be extremely high [105,107]. The difference in toxic doses between pomegranate and *T. oblongata* suggests that punicalagin potency varies between sources, and/or terminalin has synergistic effects with punicalagin causing increased potency. Despite this, pomegranate toxicity in cattle has been reported when large quantities have been available for consumption, resulting in centrilobular degeneration and necrosis [47]. Diagnostic features of punicalagin toxicity are summarized in Table 3.

#### 7.1.4. Myoporaceae

Many species of the Myoporaceae family are native and endemic to Australia. *Myoporum insulare* (Boobialla), *M. montanum* (Boobialla) and *Eremophila deserti* are known to cause hepatotoxicity in cattle and sheep [26,57]. Toxicity is due to furanosesquiterpenoid essential oils including ngaione; however, the mechanism for toxicity is unknown [57]. The clinical findings and pathological lesions tend to reflect acute hepatocellular damage [26]; diagnostic features are summarized in Table 3. There is variability in histologic lesions reported in the literature. *Myoporum insulare* toxicity as described by Jerrett and Chinnock [26] is characterized by marked periportal hepatic necrosis with pooling of erythrocytes. In contrast, a study by Allen et al. [56] found centrilobular necrosis to be the most common histologic presentation in cattle. Periportal necrosis was present only in individuals supplemented with phenobarbitone, dichlorodiphenyltrichloroethane (DDT) or high-protein diets [56]. These compounds are believed to enhance CYP450 enzyme activity and thus impact the distribution of lesions, a phenomenon that has also been demonstrated in rats [108]. The significance of these findings is unclear. Jerrett and Chinnock [26] note that although the cattle in their study were on varying planes of nutrition, all naturally occurring fatalities occurred in lactating cows on improved pastures; factors that may affect the function of CYP450 enzymes. This may indicate that natural mortalities are more likely to occur when periportal necrosis is the primary lesion, supporting the inference that periportal necrosis causes more severe clinical disease.

### 7.2. Hepatotoxicities with Unidentified Toxins

#### 7.2.1. Acute Bovine Liver Disease

Acute bovine liver disease (ABLD) is a hepatopathy affecting cattle in high rainfall areas of southern Australia, including Victoria, Tasmania and south-east South Australia, with fewer reports in New South Wales and Western Australia [27,109]. Outbreaks are seasonal, occurring in autumn and less commonly in spring (Table 3) [109,110,111]. Fodder scarcity and lactation appear to be predisposing factors due to the lack of discrimination while grazing, suggesting that the toxic material is unpalatable [111]. Acute bovine liver disease has an acute presentation, with signs of illness developing within 12 to 24 h of introduction to a new pasture and mortalities within one to two days of clinical signs developing [27,109,111]. Short-term survivors often develop photosensitization and a rapid, marked drop in milk production [27,109,111]. Due to the prevalence and severity of photosensitization in most cases, facial eczema is an important exclusion. In the reported cases, *Pithomyces chartarum* has not been present in pastures, nor have the animals had the characteristic histologic changes of biliary hyperplasia and fibrosis [27].

The etiology of ABLD is poorly understood but is generally accepted to be an unknown toxin [36]. *Cynosurus echinatus* (rough dog’s tail grass) has been present in almost all Victorian outbreaks of ABLD as dry senescent material from the previous season [111]; therefore, ABLD has been tentatively linked to the presence of this grass [36]. Additionally, *Pyrenophora* (previously *Dreschlera*) spp. of fungi have been isolated from *C. echinatus* associated with ABLD outbreaks: *Pyrenophora siccans* and *P. biseptata* have been isolated from *C. echinatus* associated with two Victorian outbreaks, while an unidentified *Pyrenophora* sp. has been isolated from a suspected outbreak in Western Australia [111]. At present, difficulties culturing *P. siccans* in significant quantities, have limited studies on the effects of and *P. biseptata* and *C. echinatus* itself [111]. Aslani et al. [36] demonstrated that extracts of *P. biseptata* agar cultures as well as extracts of *C. echinatus* had cytotoxic effects on rat hepatocytes, providing some evidence that *C. echinatus* and *P. biseptata* mycelium and spores may play a role in the hepatotoxic disease in ruminants.

Lancaster et al. [111] investigated *C. echinatus* and *P. biseptata* in vivo. The grass was harvested from properties that had experienced outbreaks in the last two years and an inoculum of *P. biseptata* was prepared using an isolate from samples collected during a severe ABLD outbreak [111]. Preparations of *C. echinatus*, oats and broth were prepared with the *P. biseptata* inoculum and fed, respectively, to three bulls for three days. Biochemical and histological analysis revealed no pathologic changes; however, the production of toxic spores at specific times during the fungus lifecycle could not be excluded [111]. At present, the role of *C. echinatus* is not clear; it may simply represent a consistent marker for climatic conditions preceding outbreaks [111]. Mycotoxins remain the primary focus of inquiry due to the epidemiology of ABLD, which supports a fungal etiology, including the seasonal occurrence and prevalence during warm, high rainfall periods; as such, the involvement of *Pyrenophora* spp. has not been excluded [111]. Currently, the primary risk factor for ABLD identified by state departments is grazing cattle on unimproved pastures with abundant dry, senescent feed and dead plant matter (Table 3) [110,112]. Current management guidelines include grazing the paddock with sheep before introducing cattle, cultivating high-risk paddocks, avoiding the use of paddocks with abundant dry material, and using a few animals to “test” for toxicity on previously toxic paddocks [112].

ABLD is known to cause periportal hepatocellular necrosis, which may extend to massive necrosis in severely affected animals [27,111]. In cattle that survive the initial insult, there is evidence of hepatocellular repair in the periportal regions [27]; however, chronic pathologic changes have not been well described. Currently, differentiation from *Myoporum* spp. toxicity can only be achieved through a thorough examination for *Myoporum* spp. around the affected paddock. It remains to be determined if there are any specific gross or microscopic lesions that may be unique to cases of ABLD; the periportal to often massive distribution of necrosis and hemorrhage may be the most dramatic and unique features of this disease. The diagnostic features of ABLD are summarized in Table 3.

#### 7.2.2. Brassicas

*Brassica* crops, including turnip (*Brassica rapa* ssp. *rapa*), rape (*Brassica napus* ssp. *biennis*), swede (*B. napus* ssp. *napobrassica*) and kale (*B. oleracea* ssp. *acephala*), introduced from Europe, North America and Asia, are now cultivated across temperate and subtropical regions of Australia and have become sporadically naturalized in southern regions [57]. These plants can cause a number of diseases in cattle and are implicated in cases of secondary photosensitization, referred to as brassica-associated liver disease (BALD) [113]. Morbidity rates and disease severity vary between outbreaks; however, severe photosensitization is a common feature and typically develops three to four days after introduction to brassica forage [113]. Hematological and biochemical changes take a number of days to develop and are characterized by marked elevations in cholestatic enzyme activities and total bilirubin and marked but often less severe elevations in hepatocellular enzyme activities [40,113]. Changes consistent with inflammation, such as a left shift neutrophilia and hyperfibrinogenemia, may also be seen [114].

Macroscopic lesions include hepatomegaly and pale-brown discoloration of the liver [113]. Despite marked elevations in enzyme activities, microscopic lesions are often subtle [113]. Key findings include cholangiectasis of small interlobular ducts, accompanied by attenuation of biliary epithelium or irregular epithelial regeneration; mild ductular reaction and mild periductular edema and fibrosis; and in some cases, obliteration of bile ducts by debris or sclerosis [40,113]. Some hepatocellular changes may be present including cell swelling, hydropic or fatty degeneration, foci of lytic necrosis and occasional binucleated cells, a normal finding in young animals or regenerating livers [54,113]. Brassica-associated liver disease is therefore easily differentiated from pithomycotoxicosis with histologic examination [113].

The toxic mechanism of BALD is currently unknown but is suspected to involve the derivatives of sulfur-containing glucosinolates (GSLs), a diverse group of stable, nontoxic compounds found within all Brassicas [115]. Following ingestion, GSLs are metabolized to volatile isothocyanates or less reactive nitriles [115]. Both are produced in the gastrointestinal tract following mastication and release of GSLs, which then encounter the myrosinase enzyme system in the saliva and rumen, leading to derivative formation [115]. Nitriles can also be produced nonenzymatically by the action of ferrous ions under weakly acidic conditions [113,115,116]. It is therefore speculated that subacute ruminal acidosis may increase the risk of BALD [113,115,116]. Additionally, the presence of certain cofactors, such as nitrile-specifier protein, favor the production of nitriles [115]. Of particular interest are the epithionitriles, derivatives of only certain GSLs, which have been shown to cause hepatic and renal lesions in rats [115]. These compounds are chemically similar to epoxides and have the potential to act as alkylating agents [114].

In 2014, a particularly large outbreak of BALD occurred in Southland and Otago, New Zealand [117]. Matthews et al. [117] found that the swedes implicated in this outbreak contained large amounts of the GSL progoitrin and subsequently investigated the toxic effects of progoitrin nitriles, 1-cyano-2-hydroxy-3-butene (CHB) and 1-cyano-2-hydroxy-3,4-epithiobutane (CHEB) via oral administration to rats [117]. All rats dosed with high doses of CHB developed randomly scattered foci of hepatocellular necrosis as well as pancreatic toxicity [117]. Rats dosed with high levels of CHEB developed multiple foci of coagulative necrosis, typically adjacent to portal triads, and nephrotoxicity [117]. Low doses of CHEB, unlike the other nitriles, had a cumulative toxic effect. Rats given subtoxic doses developed variable but mild liver lesions, including occasional hepatocellular apoptosis and necrosis and anisocytosis [117].

Investigations into other GSL derivatives have included a study of 3-butenenitrile, a nitrile derivative, which caused slightly elevated GGT activities in sheep when given orally [118]. This compound did not demonstrate hepatotoxicity in rats when given at low doses; however, there has been no further investigation of this nitrile in ruminants [117]. Another GSL, 1-Methoxy-3-indolylmethyl (1-MIM) glucosinolate, has demonstrated the ability to form DNA adducts within mouse hepatocytes, resulting in glycogen depletion and induction of p53 and p21 [119]. Interestingly, these changes were dose-dependent and most pronounced in periportal hepatocytes [119], providing some evidence that certain glucosinolates may be capable of producing periportal hepatocellular necrosis. The diagnostic features of BALD are summarized in Table 3.

#### 7.2.3. *Trema tomentosa* (Poison Peach)

*Trema tomentosa* is a native Australian species that grows at the edges of rainforest and in woodland in coastal regions of eastern Australia, northwest and central Australia [57]. Toxic and nontoxic variants are virtually identical and can coexist in small geographic areas, making it difficult to differentiate them [57]. The toxic variant contains a trematoxin and an uncharacterized hepatotoxic glycoside within its leaves [57]. Case reports of *T. tomentosa* are rare and the mechanism of toxicity is not currently known. Toxicity is more common in cattle than sheep and typically occurs when cattle have access to the edges of rainforest, particularly when conditions are dry (Table 3) [57]. *Trema tomentosa* is able to regrow rapidly after clearing and may actually outcompete other plant species after these events [57]. Clinical deterioration is rapid without the development of photosensitization [54]. Necrosis is centrilobular and identical to *Cestrum* and *Xanthium* toxicity [54]. The diagnostic features of *T. tomentosa* toxicity are summarized in Table 3.

#### 7.2.4. Argentipallium blandowskianum (Woolly Everlasting)

*A. blandowskianum* (previously *Helichrysum blandowskianum*) is a perennial herb that flowers between September to March, generally causing toxicity in summer and autumn [57]. The plant is native to South Australia and western Victoria and thrives in areas that used to grow *Eucalyptus baxteri* [57]. Cattle appear to be more sensitive to toxicity than sheep; however, previous exposure may confer some resistance to subsequent ingestion [53,57]. Toxicity is relatively uncommon and tends only to affect animals on highly infested pasture (Table 3) [53,57]. The toxic component of *A. blandowskianum* is currently unknown [57]. In addition to centrilobular damage, vascular lesions may be observed in the small arteries of various organs, characterized by swollen endothelial cells, disruption of the tunica media and vacuolation and edema of the tunica adventitia [53]. The diagnostic features of *A. blandowskianum* toxicity are summarized in Table 3.

#### 7.2.5. *Lythrum hyssopifolia* (Lesser Loosestrife)

*Lythrum hyssopifolia* is an annual herb native to eastern Australia, which is found throughout southern Australia [57]. *Lythrum hyssopifolia* grows particularly well in wet soils and crop stubbles, with toxicity most common after heavy rains [57]. Ingestion of the plant is known to cause hepatic and/or renal failure in both sheep and cattle [120]. The toxic component is yet to be definitively determined: there is suspicion for the involvement of tannins, possibly with different metabolites responsible for the hepatic and renal forms [48,121]. Toxicity is well described in sheep and typically occurs 2 to 14 days after feeding on the plant [57]. It is particularly common in sheep grazing crop stubble that is heavily contaminated with *L. hyssopifolia*, as it provides the only available green feed (Table 3) [57]. Clinical signs are typical of acute hepatotoxicity and may be accompanied by dysuria and/or hindlimb ataxia, which may persist for up to two weeks [48]. Hepatic necrosis may be centrilobular or midzonal, or less commonly, individual-cell necrosis, with multinucleate hepatocytes a relatively common finding around necrotic regions [48]. In affected kidneys, cell damage is most pronounced in the proximal tubular epithelium and tubule lumens are often filled with eosinophilic granular casts [48]. Although toxicity is poorly documented in cattle, unpublished case reports suggest gross and microscopic lesions are similar to those seen in sheep [121]. The diagnostic features of *L. hyssopifolia* toxicity are summarized in Table 3.

## 8. Application of Current Knowledge and Future Directions

A review of well-understood hepatotoxins and examination of the clinical and pathological features of lesser-known toxins allows insight into their potential mechanisms. In the case of ABLD, the periportal distribution of hepatocellular injury suggests the etiologic agent must either not require bioactivation to exert toxicity or require bioactivation by periportal-specific enzymes, the former being more common among known periportal-targeting toxins. Acute bovine liver disease appears to have a predictable histopathological phenotype with varying severity, suggesting intrinsic toxic effects that target organelles in a dose-dependent manner [14]. Lesions indicative of mutagenic or carcinogenic effects have not been identified, indicating cytosolic rather than nuclear injury; however, the pathology of chronic lesions is yet to be described, and as seen with many other examples, more than one toxic compound may be involved. The clinical acuity of ABLD, severity of clinical signs and low abundance of potential hepatotoxic material at the time of outbreaks, suggests the involvement of an extremely potent hepatotoxin. Additionally, the described climatic conditions conform with those preceding other mycotoxic hepatopathies, supporting current suspicions of mycotoxin involvement; however, further epidemiolocal, pathological and microbiological investigations are required.

Brassica-associated liver disease results in clinical and biochemical findings that reflect severe cholestasis, while histologic lesions are subtle and predominated by cholangiectasis, occasional loss and regeneration of biliary epithelium [40,113]. In regard to potential toxic mechanisms, BALD lesions suggest organelle damage leading to altered signal transduction, with cell death a variable and secondary effect of cellular dysfunction [11]. Although some GSL metabolites are potential alkylating agents, current lesion descriptions in ruminants are not consistent with mutagenic capacity, indicating primary cytosolic effects [13,15]. The development of toxin resistance in cattle that are slowly introduced to brassica forage suggests induction of enzymatic pathways that successfully detoxify metabolites. Current efforts are focused on the investigation of GSL derivatives, which appears to be a promising area of inquiry despite significant differences between brassica variants [117]. Areas requiring further examination include the role of ruminal pH and nitrile concentrations in tissues of affected cattle.

*Argentipallium blandowskianum, L. hyssopifolia* and *T. tomentosa* are poorly understood hepatotoxins due to the sporadic nature of outbreaks. In cases of *A. blandowskianum and T. tomentosa* toxicity, centrilobular hepatocellular necrosis is consistent with dose-dependent organelle damage caused by an intrinsic hepatotoxin [54]. *Argentipallium blandowskianum* toxicity also causes vascular damage suggesting endotoxic effects [54]. As with BALD, the development of resistance to *A. blandowskianum* indicates the induction of detoxifying metabolic pathways following exposure. The sporadic nature of outbreaks suggests uncommon exposure to clinically significant quantities of the plant. In contrast, *L. hyssopifolia* causes less predictable hepatic pathology. The presence of multinucleate hepatocytes suggests either antimitotic effects or incomplete cell fusion [54], the former more common in cases of hepatotoxicity [78]. Concurrent nephrotoxicity may represent the involvement of a second plant metabolite, as seen with *T. oblongata* [51,52], or spill-over of the primary toxic component or metabolites of degradative enzymatic pathways, as seen with PA, cycasin, phomopsins, amatoxins and AFB1 [4,30,77,81,90]. The varying phenotypes of zonal hepatocellular injury are similar to that of Myoporaceae toxicity and presumably influenced by microsomal enzyme depression or induction [56,108]. Considering this, the sporadic nature of outbreaks, while possibly explained by plant exposure, may equally be affected by exposure to endogenous or exogenous cofactors.

In conclusion, plant- and fungal-derived hepatotoxicities in cattle provide a diagnostic challenge to veterinarians due to the significant overlap in clinical presentations, clinical pathology and, in many instances, gross and microscopic pathology. Revision of the distinguishing characteristics highlights diagnostic features and provides insight into the potential mechanisms of lesser-known toxins. In the case of uncommon or sporadic toxicities, specific etiological investigations are required. Where this is not possible, further work on clinical, pathological and epidemiological features of these diseases is integral for their effective diagnosis and management.

## Figures and Tables

**Table 1:** Plant- and fungus-associated hepatotoxicities of cattle caused by known toxins. Summary of risk factors, histological features and distinguishing clinical features.

Plant/Fungus; Toxin	Risk Factors	Salient Macroscopic and Histopathologic Features	Salient Clinical Features
*Xanthium strumarium* (Noogoora burr); Carboxyatractyloside	Spring and summer post-rainfall	Centrilobular hepatic degeneration and necrosis	Gastrointestinal irritation
Boraginaceae, Compositae, Leguminosae; pyrrolizidine alkaloids	Reduced grazing discrimination (fodder scarcity, summer; increased energy demand, lactation, gestation)	Acute: centrilobular hepatic degeneration and necrosis Chronic: portal fibrosis, biliary hyperplasia, veno-occlusive lesions, megalocytosis	Commonly a chronic clinical course
Cycadales; cycasin	“Addicted” cattle within the group	Centrilobular hepatic degeneration and necrosis, hepatocellular megalocytosis	Dysentery; hemorrhagic necrosis of abomasal and small intestinal mucosa
*Lupinus* spp. (*Diaporthe toxica*); phomopsins A–E	Autumn occurrence (toxin accumulation begins after summer rainfall)	Individual hepatocellular degeneration and necrosis, portal fibrosis, biliary hyperplasia	Phomopsin-induced inappetence may cause an acute fatty-liver syndrome in cows during gestation or lactation
*Pithomyces chartarum* (Perennial Ryegrass); sporidesmin	Temperatures 20–25 °C, high humidity (late summer, early autumn)	Atrophy and fibrosis of the left liver lobe, portal fibrosis, cholestasis, biliary hyperplasia	Severe photosensitization, weeks to months post-exposure
*Amanita* spp., *Galerina* sp. and *Lepiota* sp. of fungi; amatoxins	-	Centrilobular to massive hepatic degeneration and necrosis, renal tubular necrosis	Low morbidity rate, peracute mortalities
*Aspergillus* spp.; aflatoxin B1	Preharvest infection of summer cereals: high temperature, low humidity Post-harvest contamination of feedstuff: 25–37 °C, high humidity	Portal fibrosis, biliary hyperplasia, hepatocellular megalocytosis	Chronic ill-thrift, extrahepatic biliary obstruction

Abbreviations: HE, hepatic encephalopathy.

**Table 2:** Plant- and fungus-associated hepatotoxicities of cattle. Summary of toxicokinetics and toxicodynamics.

Plant/Fungus; Toxin	Bioactivation	Mechanism of Toxicity	Target Organs
*Xanthium strumarium* (Noogoora burr); Carboxyatractyloside	Direct-acting	Mitochondrial ATP depletion	Liver, gastrointestinal tract, kidneys
Boragniaceae, Compositae, Leguminosae; pyrrolizidine alkaloids	Hepatic CYP450: ester and alcoholic pyrroles	Macromolecules (proteins). Nucleus: DNA cross-linking	Liver. Lesser: lungs, kidneys, placenta
Cycadales; cycasin	Small intestine: MAM, hepatic CYP450: methanol, formic acid	DNA alkylation	Liver, gastrointestinal tract, CNS. Lesser: kidneys, lungs
*Lupinus* spp. (*Diaporthe toxica*); phomopsins A-E	Direct-acting	Microtubule destruction/ inhibition of formation, inhibition of mitochondrial respiration	Liver, kidneys, adrenal glands, pancreas, rumen, reticulum
*Pithomyces chartarum* (Perennial Ryegrass); sporidesmin	Direct-acting	Oxidative injury via formation of superoxide radicals	Liver (bile canaliculi)
*Amanita* spp., *Galerina* spp. and *Lepiota* spp. of fungi; amatoxins	Direct-acting	Inhibition of protein synthesis via binding of nuclear RNAP II	Liver, kidneys
*Aspergillus* spp.; aflatoxin B1	Hepatic CYP450: epoxide	DNA alkylation	Liver, kidneys, bone marrow, lungs. Lesser: brain, muscle, adipose tissue
*Lantana camara;* lantadenes	Cecum: RLA, RLB, M1, M2	Inhibition of mitochondrial respiration, possibly other unknown mechanisms	Liver, rumen, kidneys
*Cestrum parqui* L’herit (green cestrum); carboxyparquin	Unknown	Unknown	Liver
*Terminalia oblongata, Punica granatum*; punicalagin	Unknown	Unknown	Liver
*Myoporum tetrandrum* (Boobialla); esquiterpenoid essential oils	Unknown	Unknown	Liver, kidneys, gastrointestinal tract. Lesser: adrenal glands
ABLD (unknown); unknown	Unknown	Unknown	Liver
*Brassica* spp.; unknown	Unknown	Unknown	Liver
*Trema tomentosa* (poison peach); unknown hepatotoxic glycoside	Unknown	Unknown	Liver
*Argentipallium blandowskianum* (woolly everlasting); unknown	Unknown	Unknown	Liver, kidneys, lungs, heart, skin, spleen and gastrointestinal tract
*Lythrum hyssopifolia* (lesser loosestrife); unknown	Unknown	Unknown	Liver, kidneys

Abbreviations: RLA, reduced lantadene A; RLB, reduced lantadene B; RNAP II, RNA polymerase II; MAM, methylazoxymethanol; CYP450, cytochrome P450 monooxygenases; ABLD, acute bovine liver disease.

**Table 3:** Plant- and fungus-associated hepatotoxicities of cattle caused by unidentified toxins or toxins with unidentified mechanisms. Summary of risk factors, histological features and distinguishing clinical features.

Plant/Fungus; Toxin	Risk Factors	Salient Macroscopic and Histopathologic Features	Salient Clinical Features
*Lantana camara;* lantadenes	Reduced grazing discrimination (fodder scarcity, summer; increased energy demand, lactation, gestation)	Hepatocellular megalocytosis, cholestasis, biliary hyperplasia and fibrosis. Degeneration of proximal tubular epithelium, cystic distension of tubules	Ruminal stasis and constipation
*Cestrum parqui* L’herit (green cestrum); carboxyparquin	Reduced grazing discrimination (fodder scarcity, summer; increased energy demand, lactation, gestation)	Centrilobular hepatic degeneration and necrosis	Ruminal stasis and constipation
*Terminalia oblongata, Punica granatum* L.; punicalagin	-	Centrilobular hepatic degeneration and necrosis	Gastrointestinal irritation
*Myoporum tetrandrum* (Boobialla); furanosesquiterpenoid essential oils	-	Periportal or centrilobular hepatic degeneration and necrosis, depending on CYP450 activity	-
ABLD (unknown); unknown	Unimproved pastures with senescent plant material, autumn (occasionally spring)	Periportal to massive hepatic degeneration and necrosis	Acute onset
*Brassica* spp.; unknown	Rapid introduction to *Brassica* forage	Subtle histologic changes, cholangiectasis of small ducts, cholangiocyte attenuation and regeneration	-
*Trema tomentosa* (poison peach); unknown hepatotoxic glycoside	Reduced grazing discrimination (fodder scarcity, summer; increased energy demand, lactation, gestation)	Centrilobular hepatic degeneration and necrosis	-
*Argentipallium blandowskianum* (woolly everlasting); unknown	Summer and autumn	Centrilobular hepatic degeneration and necrosis, endothelial cell degeneration and perivascular edema	-
*Lythrum hyssopifolia* (lesser loosestrife); unknown	Post heavy rains, grazing heavily contaminated crop stubble	Centrilobular, midzonal or individual hepatocellular necrosis, multinucleated hepatocytes, proximal tubular epithelial necrosis	Dysuria and/or hindlimb ataxia occasionally present

Abbreviations: ABLD, acute bovine liver disease; CYP450, cytochrome P450 monooxygenases.
